# Extensive Testing May Reduce COVID-19 Mortality: A Lesson From Northern Italy

**DOI:** 10.3389/fmed.2020.00402

**Published:** 2020-07-14

**Authors:** Mauro Di Bari, Daniela Balzi, Giulia Carreras, Graziano Onder

**Affiliations:** ^1^Research Unit of Medicine of Aging, Department of Experimental and Clinical Medicine, University of Florence, Florence, Italy; ^2^Unit of Geriatrics, Department of Medicine and Geriatrics, Azienda Ospedaliero-Universitaria Careggi, Florence, Italy; ^3^Unit of Epidemiology, Department of Prevention, Azienda USL Toscana Centro, Florence, Italy; ^4^Department of Cardiovascular, Endocrine-Metabolic Diseases and Aging, Istituto Superiore di Sanità, Rome, Italy

**Keywords:** COVID-19, epidemics, surveillance, swab testing, mortality, echologic studies

## Abstract

The effects of different COVID-19 swab testing policies in Italy need investigation. We examined the relationship between the number of COVID-19 swab tests (per 10,000 population) performed from February 24 through March 27 and 7-day lagged COVID-19 mortality (per 10,000 population) in four regions of northern Italy. Lombardy, Piedmont, and initially, also Emilia-Romagna, which followed recommendations for limiting swab testing to symptomatic subjects requiring hospitalization, had a much steeper increase in mortality with increasing number of tests performed than Veneto, which applied a policy of broader testing. The relationship between tests performed and mortality declined in Emilia-Romagna in coincidence with a substantial increase in the number of tests performed on March 18. When the cumulative number of tests performed was regressed linearly toward lagged mortality in Lombardy and Veneto, the slope of the regression was 133 in Veneto and 10.4 tests per one death in Lombardy. These findings suggest that the strategy adopted in Veneto, similar to that in South Korea, was effective in containing COVID-19 epidemics and should be applied in other regions of Italy and countries in Europe.

## Introduction

On February 20, 2020, a first autochthonous case of COVID-19 respiratory disease was observed in Lombardy, Italy (1), soon followed by a second patient in Veneto. Since then, the outbreak has rapidly expanded, mostly in regions in northern Italy (2), with unprecedented violence. For many weeks, Italy has been the Western country with the highest incidence of, and grimly also with the greatest death toll from, COVID-19.

Initially, epidemiological surveillance and strategies for swab testing, followed by COVID-19 reverse transcriptase-polymerase chain reaction (RT-PCR), were under the control of regional healthcare authorities. On February 25, the Italian Ministry of Health issued more stringent policies for swab testing, prioritizing symptomatic patients with possible COVID-19 contacts requiring hospitalization. Most regions complied with these recommendations, whereas Veneto maintained its policy, implemented after the occurrence of the first cases, of extensive testing and isolation of positive cases (3). Surprisingly, the debate stemming from these different regional policies valued international more than Italian evidence (4). We aimed at assessing, using data from the first month of the Italian experience, how different policies for swab testing may impact on the initial progression of COVID-19 epidemics.

## Methods

Data were obtained from the publicly available reports issued by the Italian Department of Civil Protection (5), basically containing the numbers of swabs, active cases, patients admitted to hospital and intensive care units, and deaths. We compared Lombardy, Emilia-Romagna, and Piedmont, regions in northern Italy that closely followed the recommendations for restrictive COVID-19 testing, and Veneto, which applied a policy of broader testing (3).

The cumulative number of RT-PCR tests performed from February 24 through March 27 and COVID-19 cumulative mortality from March 2 through April 3 were indexed by population in each region (6). A 7-day lag time between COVID-19 testing and mortality was allowed because death usually occurs 7+ days after clinical onset and diagnosis (2).

Piecewise linear regression was applied, separately for the four regions, to identify the breakpoints in the slope of the number of tests performed over time and to examine whether the relationship between the cumulative number of tests performed through each date (independent) and mortality (dependent variable) followed a different progression over time in the four regions.

The effectiveness of the two testing strategies was estimated by regressing the number of tests (dependent) and the cumulative lagged mortality (independent variable), separately for the two most distant scenarios of Lombardy and Veneto: the slope of these regressions represents the number of tests associated with one death.

To compensate for delays and imprecisions in the daily reporting of data, the proportion of positive cases was calculated as the percent ratio of the 3-day moving averages of positive cases over tests performed. Differences in the proportion of positive cases across the four regions were assessed with one-way ANOVA and the Games Howell test for unbalanced variances for *post-hoc* comparisons. Pearson's *r* correlation coefficient was used to assess whether the proportion of positive tests changed with the cumulative number of tests performed.

Data management and statistical analyses were conducted with IBM SPSS®, version 26.0, and R version 3.5.3 (package segmented). Protection against type I error was set at a = 0.05.

## Results

**Figure 1:**
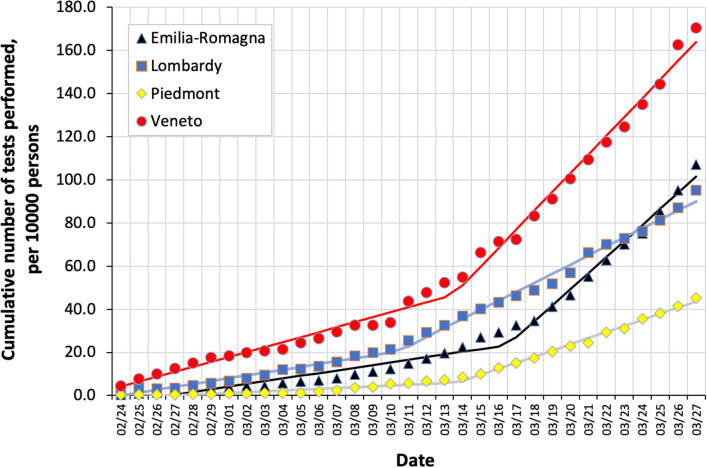
Cumulative number of COVID-19 tests performed in four regions in northern Italy from February 24 through March 27, per 10,000 persons in each region. Piecewise regression lines are also shown.

The number of tests increased daily from February 24 through March 27 in all regions, although with marked differences: starting from 0.3, 1.5, 0.3, and 4.5 tests per 10,000 persons by February 24, Emilia-Romagna, Lombardy, Piedmont, and Veneto reached 107.2, 95.3, 45.2, and 170.5 tests per 10,000 persons by March 27 (Figure 1). Piecewise regression showed that the progressive increase in the number of tests performed changed slope in all regions with different timing and extension: the slope increased by March 10 in Lombardy, March 14 in Veneto and Piedmont, and March 17 in Emilia-Romagna, being initially lowest in Piedmont, intermediate in Lombardy and Emilia-Romagna, and maximal in Veneto (0.4, 1.3, 1.2, and 2.3 more tests per 10,000 per day, respectively). It then increased to a similar extent in Veneto and Emilia-Romagna, although it remained smaller in Lombardy and Piedmont than in the other two regions (8.7, 7.4, 4.2, and 2.8 more tests per 10,000 per day, respectively).

**Figure 2:**
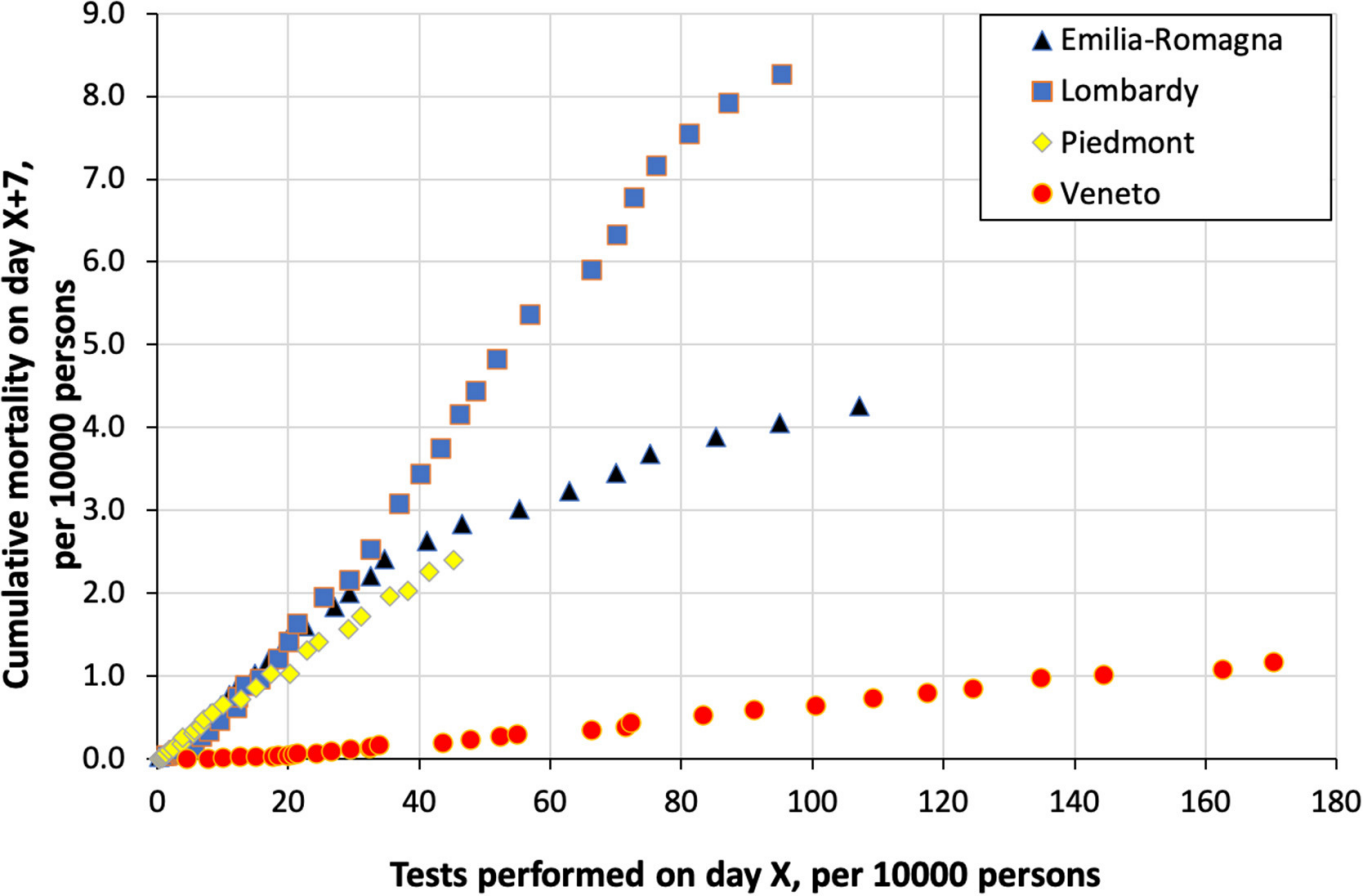
Cumulative COVID-19 mortality in four regions in northern Italy from March 2 through April 3 as a function of the cumulative number of COVID tests performed 7 days before, i.e., from February 24 through March 27.

From March 2 through April 3, COVID-19 mortality increased from 0.02, 0.04, 0.00, and 0.004 to 4.27, 8.26, 2.39, and 1.17 per 10,000 persons in Emilia-Romagna, Lombardy, Piedmont, and Veneto, respectively. Figure 2 shows the relationship between the cumulative number of COVID-19 tests performed from February 24 through March 27 and the corresponding lagged mortality (March 2-April 3) in the four regions. Compared to Veneto, Lombardy, Piedmont, and, until March 17, also Emilia-Romagna clustered toward a steeper mortality rate increase with increasing number of tests performed. After that date, in Emilia-Romagna, the slope of the relationship flattened substantially: the piecewise regression confirmed a change in the slope when the cumulative number of 41.1 tests per 10,000 was reached, in coincidence with the sudden increase in the rate of daily testing on March 18. Slopes remained unchanged in the other regions. When the cumulative number of tests performed was regressed linearly toward lagged mortality in Lombardy and Veneto, the slope of the regression was 133 in Veneto and 10.4 tests per one death in Lombardy.

The proportion of positive tests was (mean ± SD) 26.8 ± 9.4, 33.7 ± 13.1, 28.7 ± 12.3, and 8.0 ± 3.2 percent in Emilia-Romagna, Lombardy, Piedmont, and Veneto, respectively (*p* < 0.001), with significant differences (*p* < 0.001) between Veneto and each of the other regions on *post-hoc* comparisons. The proportion of positive tests decreased with the number of tests performed in Emilia-Romagna (*r* = −0.381, *p* = 0.017), remained unchanged in Lombardy (*r* = 0.164, *p* = 0.318) and Veneto (*r* = 0.184, *p* = 0.262), and increased in Piedmont (*r* = 0.341, *p* = 0.034).

## Discussion

We observed that extensive swab testing, applied since the beginning of the epidemics, may contribute to reducing the spread of COVID-19 by identification of a high number of positive cases that can eventually be isolated (3, 7). Four regions in the same area of Italy, almost simultaneously hit by the virus, adopted different strategies for COVID-19 outbreak containment. In Veneto, where a policy for extensive testing followed by strict isolation of positive cases was applied (3), the increase in COVID-19 mortality was milder than in the other regions, which initially clustered in a steeper relation between the number of tests and mortality. Accordingly, the proportion of positive tests was lower in Veneto than elsewhere, whereas the rate of daily increase in mortality in Emilia-Romagna, initially similar to that in Lombardy, declined when the rate of daily increase in the number of tests performed became steeper. Thus, whereas with its policy of extensive testing, Veneto was efficaciously containing the spread of the disease, Lombardy, Piedmont, and initially also Emilia-Romagna, were rather chasing the virus, using tests more to confirm clinically plausible diagnoses than to contain the epidemics.

We estimated the spread of the disease using COVID-19 mortality instead of the number of positive tests, which depends heavily on the policy for testing: other factors being the same, the broader the criteria for testing, the wider the denominator, the lower the proportion of positive tests, and vice versa. A 7-day lag was allowed to identify deaths, as this is the minimum interval to attribute death to COVID-19 (2).

In current times, no epidemics have spread throughout the world with an extent and virulence similar to COVID-19. Italy is no exception. The closest comparison might be with the H1N1 swine flu pandemic in 2009: nevertheless, that was a definitively milder disease, responsible for only 260 deaths in Italy (8), approximately 130 times less than those directly attributed to COVID-19. Thus, the challenges posed by COVID-19, including those referring to containment measures, cannot be reasonably compared to any prior epidemics.

Using publicly available data (9), it can be observed that South Korea was the first in the world to begin to apply massive COVID-19 swab testing as a premise to the isolation of positive cases and quarantine of suspected cases. It did so as early as January 21, when only a few cases had been recorded in the country. This policy was subsequently maintained, and a similar approach was followed by New Zealand a few weeks later: as of May 13, these two countries had performed as many as 132 and 434 tests per 10,000 persons, respectively. The number of tests performed in Italy by the same date was of comparable magnitude (453 per 10,000), but the total number of confirmed cases (3,659 per million) was more than 10 times higher, and that of deaths (511 per million) 100 times greater, than in South Korea (214 and 5.1 per million) and New Zealand (238 and 4.4 per million).

Our study is limited by its ecological-type design, which does not allow the consideration of other variables (including individual susceptibility to more severe forms of the disease or systemic factors, such as healthcare response) that may play a role in the relationship between testing policy and COVID-19 mortality. Moreover, the validity and generalizability of our findings depend on the quality of the source database, where issues around the accuracy and timing of reporting have been raised (10).

Being the first western country to face COVID-19 outbreak, Italy represents a living laboratory in which to evaluate the effectiveness of practices to combat it (11). Other authors have shown that the lockdown measures enforced by the Italian government had a measurable impact on the progression of COVID-19 epidemics, supporting WHO recommendations for strict containment measures as early as possible in the epidemic curve (12). Our findings echo those from international comparisons, indicating that a broader policy for swab testing, such as that applied in Veneto, may contribute to containing COVID-19 threat.

## Code Availability

IBM SPSS®, version 26.0, and R version 3.5.3 codes are available from authors on request.

## Data Availability Statement

The raw data supporting the conclusions of this article will be made available by the authors, without undue reservation.

## Author Contributions

MD and DB conceived the study. MD and GC performed statistical analyses. MD drafted the manuscript, which was revised by all other authors. GO provided institutional support. All authors contributed to the article and approved the submitted version.

## Conflict of Interest

The authors declare that the research was conducted in the absence of any commercial or financial relationships that could be construed as a potential conflict of interest.
